# Efficacy of Topical Steroid Ointment in Treating Phimosis: A Review of Clinical Practice

**DOI:** 10.7759/cureus.88130

**Published:** 2025-07-16

**Authors:** Patrick A Dewan

**Affiliations:** 1 Pediatric Surgery, Pediatric Urology Unit, Northern Private Hospital, Melbourne, AUS

**Keywords:** circumcision, foreskin, phimosis, prepuce, steroid treatment

## Abstract

Background: Topical steroids as first-line treatment for phimosis are well established, but both the accurate diagnosis of pathological phimosis and the regimen of use of steroid ointment are not widely known, as judged by a review of referrals to a specialist clinic.

Objective: This study aims to determine the proportion of patients with an accurate diagnosis of phimosis and appropriate use of steroid treatment prior to specialist referral for phimosis.

Materials and methods: Retrospective data were collected for 781 boys with a mean age of 11.8 ± 0.22 years, including the reason for general practitioner referral, previous use of topical steroids, and specialist management.

Results: Six hundred patients were referred with a diagnosis of phimosis; 181 were referred with an alternative diagnosis. Only 218 boys had pre-referral steroid treatment. Conservative therapy was used for 614 patients, of whom 489 received topical steroid ointment. Circumcision or dorsal slit procedures were required for failed medical management in 95.

Conclusions: Most boys with phimosis are cured with the use of an appropriate topical steroid; however, very few have steroid ointment as part of their care prior to referral.

## Introduction

“Phimosis” is defined as the inability to retract the prepuce over the head of the penis (glans), specifically because of foreskin narrowing, rather than adhesion of the inner layer of the foreskin to the glans [1], noting that phimosis is a common presentation to the pediatric urology services in a predominantly uncircumcised community. Most cases result in circumcision [2].

Penile development occurs from the seventh week of gestation and is completed by the 17th week. The outer layer of the penis folds on itself to form the prepuce, which covers the glans, including the urethral meatus. The function of the prepuce is protective, immunological, and erogenous (new reference).

At birth, 96% of males have the inner foreskin adhered to the glans; thus, the foreskin is not retractable; to reiterate that non-retractability because of adhesions is not phimosis [1].

Physiological (congenital) phimosis is when the distal foreskin is narrow and retracts to some extent in the process of gentle unrolling, with an appearance akin to a rosebud opening. Pathological phimosis is a non-retractable foreskin with a usually tight, scarred, dome-shaped foreskin with sometimes only a pinhole opening. There may also be secondary balanitis xerotica obliterans (BXO) changes.

Diagnosis of phimosis is predominantly clinical, and no imaging studies or laboratory tests are typically required. However, a midstream specimen of urine and ultrasound may be indicated if an infection has occurred.

Traditionally, circumcision has been the surgical treatment for phimosis. However, there are other surgical options, and many pathological phimosis cases can be cured using topical steroid ointment if combined with physical therapy.

## Materials and methods

Data were retrospectively collected from 781 patients who attended a single-practitioner pediatric urology clinic in Melbourne, Australia. Most of the patients were young boys; a few were adults at the time of presentation, with an age range of two months to 48 years and a mean age of 11.8 ± 0.22 years.

Notes were reviewed for all patients in the clinic, with those with management of their foreskin retrieved from the files for evaluation of demographic data and details of the findings and management of their foreskin.

In addition to the 600 referred for management of phimosis, another 181 were sent for a specialist opinion to rule out alternative pathologies, and during a thorough examination, they were found to have phimosis. The notes were retrieved and examined with a preset list of information to be recorded, including the nature of the presentation and referral, details therein, the pre-referral treatment, the specialist examination findings, the management, and the outcome for the care of the foreskin after the specialist referral.

In particular, the date of birth and presentation were recorded for all patients treated over 10 years. Additionally, whether a steroid ointment had been used prior to referral and the type of steroid used were considered of particular importance and were noted. For those found to have pathological phimosis that appeared to be remediable by medical management, the patient had a four-week course of betamethasone twice daily and was then reviewed. Further follow-up and the outcome at surgery (if it occurred) were also recorded in the Excel data spreadsheet (Microsoft Corp., Redmond, WA, USA).

Review of the notes resulted in the direct entry of de-identified information into a purpose-designed Excel spreadsheet, from which the interpretation was facilitated by tabulation and analysis.

## Results

Phimosis was diagnosed in 600 boys by their general practitioner (GP) and another 181 were referred with an alternative diagnosis and found to have phimosis; the latter group included 33 with renal pathology; and phimosis was diagnosed in 21 boys who were referred for an elective circumcision, with the remaining 127 boys having a range of unrelated pathologies, including undescended tests (Table 1). Of the 600 who were referred to paediatric urologists with phimosis, 92 were found to have a normal foreskin, and 15 were found to have resolved narrowing after the referral had been instigated. Pre-referral GP treatment was with steroid ointment in only 218 boys; 88 were prescribed a potent steroid, 92 a low or moderate potency steroid, and in 38, the potency was not recorded in the record (Table 2).

**Table 1:** GP referral diagnosis GP: general practitioner

Findings	Frequency	Percentage
Phimosis	600	76.8
Non-renal	148	19.0
Renal	33	4.2
Total	781	100.0

**Table 2:** Pre-referral treatment

Findings	Frequency	Percentage
More than 1 steroid	2	0.3
Antibiotic ointment	2	0.3
None	559	71.6
Steroid ointment	218	27.9
Total	781	100.0

Table 3 shows the management by the specialist, conservative treatment being the mainstay of therapy, which was used for 614 patients, of which 489 received topical steroid ointment. Circumcision or dorsal slit procedures were required for failed medical management of phimosis in 95; another 72 patients also had a surgical procedure for management of their foreskin as the parental preference; most had a circumcision. For 127 boys, the only treatment was physical therapy for the foreskin, namely retraction with nappy changes or voiding, and at the time of bathing. Physical therapy and steroids were used in 471 boys, and 15 were discharged without any treatment as they had a normal foreskin and their family did not elect to have a circumcision.

**Table 3:** Specialist management

Findings	Frequency	Percentage
Circumcision	46	5.9
Failed steroid + circumcision	81	10.4
Dorsal slit procedure	8	1.0
Failed steroid + dorsal slit	14	1.8
Physical therapy only	127	16.3
Steroid + physical therapy	471	60.3
Steroid success + elective circ	18	2.3
Discharged	15	1.9
Other	1	0.1
Total	781	100.0

Table 4 shows the specialist's treatment and outcome for 781 patients. Initially, 93 were normal at presentation, and in 15, the phimosis had resolved. In addition to those who had an accurate referral diagnosis of phimosis, 21 were referred for elective circumcision by the GP, and 159 were found to have phimosis after being referred for another reason.

**Table 4:** Specialist diagnosis

Findings	Frequency	Percentage
Initially normal	93	11.9
Previously resolved	15	1.9
Phimosis	667	85.4
Other	6	0.8
Total	781	100.0

## Discussion

This study aimed to identify the proportion of patients with an accurate diagnosis of phimosis when referred to a pediatric urologist by their GP. Additionally, the retrospective data were used to evaluate the frequency of appropriate management with steroids prior to specialist referral. Topical application of steroids is considered an appropriate means of management of phimosis, noting the prospective study by Orsola et al., who reported topical steroids to be safe, inexpensive, and effective in the management of phimosis [3].

Phimosis is defined as the inability to retract the prepuce due to the narrowing of the foreskin [2]. At birth, the foreskin is usually not retractable as the inner epithelial surface is fused to the glans; thus, preputial adhesions are a normal part of the development of the foreskin [4]. In 96% of males, the prepuce becomes retractable to expose the urethral meatus and a part of the glans by the age of 3 [5].

Accurate diagnosis of phimosis is crucial, as it enables the provision of appropriate treatment. When examining the patient, scarring of the preputial ring may be observed. Patients presenting with this condition can vary in severity, which can be categorized into four grades. Grade I involves a fully retractable prepuce with a stenotic ring that can be observed along the shaft; grade II allows partial exposure of the glans with partial retractability of the prepuce. Grade III severity will also allow for partial retractability of the prepuce; however, only the meatus can be exposed. The most severe grade IV presentation prevents the prepuce from being retracted [1]. It should be noted that a fully adherent normal prepuce, when retracted, may only allow visualization of the meatus as the glans is fully covered by adherent tissue in the layer of the prepuce.

Balanitis (inflammation of the glans penis) was present in 6.6% of the patients referred to the pediatric urologist. A more severe form of balanitis, known as BXO, or lichen sclerosus, is an unusual condition that can cause hyperkeratosis and hyperplasia of the squamous mucosa and homogeneous collagen deposition in the dermis of the penile skin [6]. Symptoms can include white patches or spots on the foreskin. Thirteen percent of our cohort were found to have BXO.

There are various treatment options available for managing phimosis [7]. A patient with phimosis does not always require treatment; physiological phimosis may be managed with appropriate physical care and is a normal phenomenon that resolves over time, typically identifiable by the pouting of the non-scarred distal prepuce. Most boys are born with some adherence to the inner layer of the foreskin, which is different from phimosis, and the adhesions separate naturally over time.

Most cases of pathologic phimosis can be treated with physical therapy alone or with the addition of steroid ointment. The concept of physical therapy for the foreskin involves tissue expansion through gentle retraction, which increases the circumference of the phimotic band. When skin undergoes gradual mechanical stretching, both passively and actively, it stimulates the formation of new cells through the process of differentiation and mitosis.

Based on the findings from the collected data, it is evident that GPs referred 600 patients to the specialist with a diagnosis of phimosis. However, the specialist documented that only 81% of these patients had an accurate diagnosis, with about 15% having normal findings, and the remaining few probably had resolution of phimosis before specialist consultation. Nevertheless, it is important to identify that 23% of the total patients had missed the diagnosis of phimosis, who had been referred for elective circumcision or had an alternative diagnosis. A possible factor that may have contributed to these missed diagnoses is that a thorough physical examination was missed. The use of steroid ointment for phimosis makes up a significant portion of the management of the condition, which needs to be implemented by primary care physicians following diagnosis. It is possible that GPs may or may not be aware of the appropriate management for this condition. Despite this assumption, a significant proportion, i.e., 72%, of boys had been referred to the specialist without steroid management.

Steroid ointments for the treatment of phimosis are used by applying them at the tip of the foreskin, making stretching easier. On using it for two weeks, it allows the child or their parent to begin physical therapy with gradual stretching, which generally involves sliding the foreskin as far as possible without causing pain. Gentle stretching is important, as application of force can both hurt the child and damage the foreskin. Given adequate retraction is possible, the steroid may also be applied along the exposed portion of the glans, following which the prepuce is allowed to slide back to its normal position. Different types of topical steroids can be used for the management of this condition, which include betamethasone, clobetasol, mometasone furoate, sodium diclofenac, and triamcinolone acetonide [8-15].

Based on previous studies, betamethasone (among other steroids) has been shown to have the best improvement rates for treatment [8]. A study conducted by Marques et al. demonstrated that the use of 0.05% betamethasone ointment produced a success rate of 94.2% among patients who had a stenosed foreskin with a varying degree of exposure of their glans [5]. Other studies have shown similar success rates [9-15]. They explored the effectiveness of the ointment by prescribing betamethasone to these patients for a minimum period of 30 days and a maximum of four months. Although many reports discuss the application of an ointment for one month, it was found that the desired outcome may take several months.

Following referral to the specialist, 75% of the patients had undergone steroid management, which their GPs could have easily commenced. It is not clear why GPs have avoided the use of steroid ointment (which would need to be investigated further). However, it is important that they are prompted to carry out a thorough physical exam and educated about the importance of steroid management among patients with phimosis. Also, there is variability in the potency of steroids used by GPs, with only 40.4% (88) of 218 cases treated with adequate steroids, which would be a point that could be highlighted in educational material.

Based on the findings, 81% (487) of the 600 patients referred by GPs with phimosis had foreskin narrowing, 15% (92) had a normal foreskin, and 2.5% (15) had resolved phimosis by the time of the specialist review. Notably, 23% (181) of the 781 patients have phimosis identified when presenting for elective circumcision in 21 cases and for alternative reasons in 160 patients, most of whom had either an inguinal hernia or undescended testes. A possible factor that may have contributed to these missed diagnoses is that a thorough physical examination was missed.

We recognized that one limitation of our study is the lack of extensive clinical detail in the referral letters. Noting that, exhaustive information would only be achieved by photographic evidence being routinely collected by GPs prior to referral. Which is not feasible.

## Conclusions

Our study had shown that most boys with phimosis are cured with the use of an appropriate topical steroid, provided foreskin physical therapy is included in the management. Our review has shown that the appropriate steroid ointment has not been used in a significant proportion of boys prior to referral to a specialist, and those with normal adhesions of the inner layer of the foreskin are also referred, inappropriately, with a diagnosis of phimosis.

By highlighting the outcome of this review, we hope to empower GPs to assist their communities by gently unrolling the non-inflamed prepuce during examinations, using steroid ointment when there is phimosis without BXO, and referring boys urgently when there is an obstructive component to the foreskin narrowing. We also wish to educate families to proactively care for the foreskin of an uncircumcised boy.
